# Brillouin Zone Folding‐Driven Topological Band Flips Based on BICs via Twisted Photonic Crystals

**DOI:** 10.1002/nap2.70164

**Published:** 2026-06-09

**Authors:** Guanhua Yang, Tian Sang, Leyi Zhu, Chenyang Wang, Yueke Wang

**Affiliations:** ^1^ Department of Optoelectronic Information Science and Engineering School of Optoelectronic Information and Physical Science Jiangnan University Wuxi China; ^2^ Jiangsu Provincial Research Center of Light Industrial Optoelectronic Engineering and Technology Jiangnan University Wuxi China

## Abstract

Brillouin zone folding (BZF) associated with bound states in the continuum (BICs) has provided promising opportunities for light wave manipulation. Despite its significance, achieving and understanding BZF‐induced band flips based on BICs in momentum space remain unresolved. Here, we demonstrate that such BZF‐driven topological band flips can be realized by using twisted photonic crystals (PhCs) based on BICs. The twist perturbation folds all high‐symmetry points of the bands to the Γ point, and each band generates three new bands linked to either a BIC or a guided‐mode resonance (GMR) due to the shrinkage of the first Brillouin zone (FBZ). The interaction among these BZF‐driven bands can be controlled by tuning the twist angle. At a critical angle, the degeneracy of the *p*‐like and *s*‐like bands at the Γ point creates an accidental‐degeneracy‐induced Dirac cone (ADC). Breaking this ADC by further twisting directly leads to topological band flips, evidenced by a parity exchange and a transformation between GMR and BIC modes. Experimental observations of this twist‐angle‐tailored band flipping associated with an ADC show excellent agreement with theoretical simulations. Our findings offer a new perspective on momentum‐space topological band engineering and pave the way for advanced light manipulation and tailorable light–matter interactions.

## Graphical abstract

Brillouin zone folding (BZF)‐driven topological band flips are demonstrated via twisted photonic crystals (PhCs) incorporating bound states in the continuum (BICs). Twist perturbation generates three new bands, each linked to either a BIC or a guided‐mode resonance (GMR). An accidental triple degeneracy forming a Dirac cone is achieved at a critical twist angle, and the twist‐tuned band‐inversion process is experimentally observed.

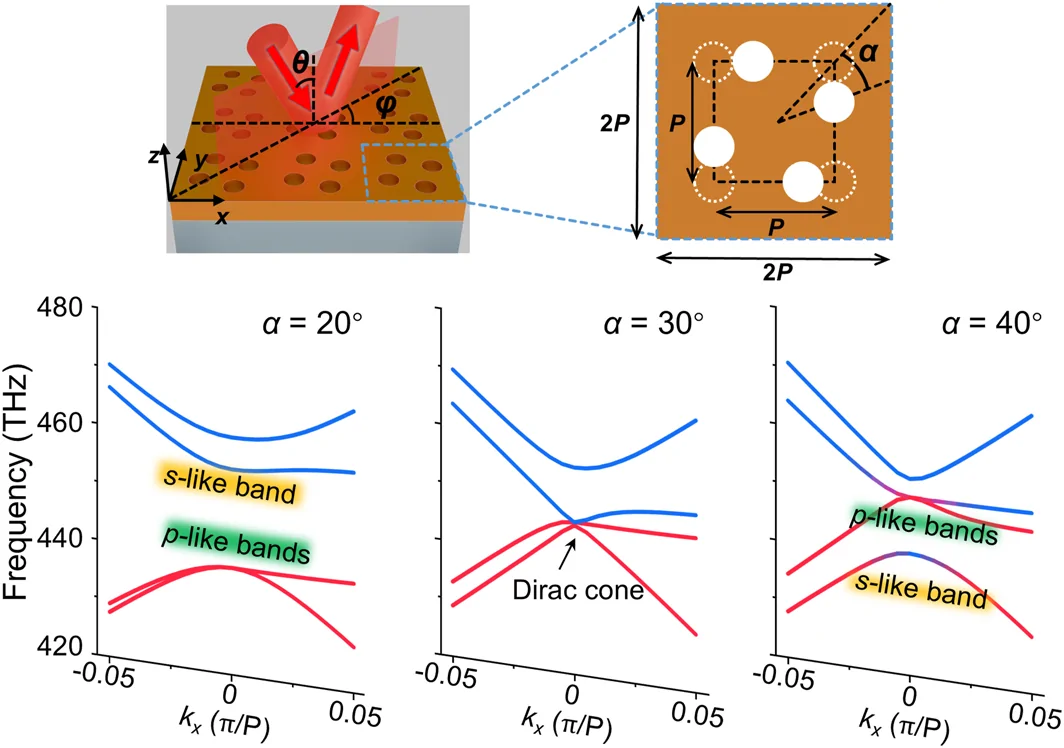

## 1 Introduction

The confinement of light in subwavelength structures with ultralong radiative lifetimes is critically important for a wide range of nanophotonic devices [1, 2]. Unlike conventional approaches that rely on mirrors or photonic bandgaps, optical bound states in the continuum (BICs) provide a distinct mechanism for perfect light trapping [3, 4]. Although embedded within the radiation continuum, BICs support modes with infinite radiative lifetimes and are thus distinct from guided modes (GMs) based on total internal reflection (TIR) [5, 6]. Two primary mechanisms are known to realize such nonradiative BICs. The first involves symmetry‐protected BICs (SP‐BICs), where eigenmodes are decoupled from the radiation continuum due to structural symmetry [7, 8, 9]. The second arises from accidental BICs (A‐BICs), achieved by tuning system parameters to induce destructive interference among multiple leakage channels [10]. In realistic optical systems, however, ideal BICs with infinite quality (*Q*) factors are not directly observable in the far field and correspond to resonances with zero linewidth in scattering spectra. By contrast, quasi‐BICs (QBICs) exhibit finite yet ultrahigh *Q* factors, enabling efficient light–matter interactions via radiative channels coupled to free space [11, 12, 13]. Benefiting from drastically enhanced light confinement, QBICs have emerged as a versatile platform for advancing nanolasers [14, 15], nonlinear optics [16, 17], sensing [18, 19], and polarization control [20].

From the perspective of topological photonics, the nonradiative nature of BICs stems from their topological far‐field polarization properties. In this context, BICs are identified as polarization singularities (V points) in momentum space, which correspond to the vortex centers of the polarization fields with integer topological charges [21]. Subsequent experimental studies further confirmed that a BIC exhibits a topological vortex in momentum space [22, 23], and optical vortices can be generated in the spatiotemporal domain by transforming a BIC into a QBIC mode [24]. Furthermore, the existence of high‐order topological charges has been observed in high‐symmetry groups or higher energy bands [25, 26], which can be tuned to merge or split by varying structural parameters [25, 26, 27]. The manipulation of topological charges is possible both within the same energy band and across degenerate bands [28]. One remarkable feature is that other polarization singularities, such as circular polarization points (C points), can be generated through V points—a process governed by global topological charge conservation [29, 30]. By designing topological polarization singularities in an on‐demand manner, intriguing phenomena and applications can be achieved, including intrinsic circular dichroism [31, 32], topological optical forces [33], chiral emission [34, 35], and unidirectional guided resonances [36, 37]. On the other hand, recent studies show that Brillouin zone folding (BZF) can be used to create BICs through periodic perturbations in metasurfaces [38]. This mechanism folds GMs, originally below the light line, into the radiation continuum. Therefore, tailoring the band structures of metasurfaces via BZF enables the realization of high‐*Q* resonances [39, 40, 41], chiroptical resonances [42, 43], and coherent thermal emissions [44, 45]. However, previous studies on BZF metasurfaces have primarily emphasized band folding characteristics along a specific direction in momentum space. The evolution of topological polarization singularities mediated by BZF across all high‐symmetry directions remains underexplored. In particular, the topological band flips associated with BICs within this framework remain an open question, despite their potential relevance to applications such as light trapping [46, 47], optical filters [48, 49, 50], nonlinear enhancement [51, 52], and quantum photonics [53].

In this work, for the first time, we report the realization of BZF‐driven topological band flips with an intermediate state of Dirac cone using twisted photonic crystals (PhCs) incorporating BICs. The introduced twist perturbation folds all high‐symmetry points of the bands of the unperturbed TiO_2_ PhC to the Γ point. This shrinkage of the first Brillouin zone (FBZ) across all high‐symmetry directions causes each original band to generate three new bands, each linked to a BIC or a guided‐mode resonance (GMR). This contrasts with previous studies, where FBZ shrinkage along a specific direction generates only one or two new bands. The twist angle enables dynamic control over the interactions among these bands induced by FBZ folding. At a critical angle, an accidental degeneracy between the *p*‐like (TE_11_ and TE_12_) and *s*‐like (TE_21_) bands at Γ creates an accidental Dirac cone. Further twisting breaks this Dirac cone associated with a triply degenerate point at the Γ point, directly triggering the topological band flips. This transition is evidenced by a parity exchange and a mode transformation between GMR and BIC modes within the TE_11_ and TE_21_ bands. This twist‐angle‐dependent band‐flipping process is experimentally observed and shows excellent agreement with theoretical simulations.

## 2 Principle of BZF via Twisted PhC

Figure 1a illustrates the schematic diagram of a twisted TiO_2_ PhC slab with thickness *h* supported by a SiO_2_ substrate. The radius of the air hole of the PhC is *r*, and the period of the primitive unit cell is *P*. The structural parameters are as follows: *h* = 100 nm, *r* = 45 nm, and *p* = 200 nm. The TiO_2_ thin film [54] and SiO_2_ substrate [55] can be regarded as lossless media in the visible region, and their refractive indices are 2.35 and 1.47, respectively. The enlarged view indicates that the periods of the primitive PhC in both the *x* and *y* directions are doubled as the air holes are twisted by an angle of *α*. Numerical simulations were performed using the commercial software COMSOL Multiphysics to investigate the eigenmode properties of PhCs. Figure 1b shows the transverse electric (TE) band structures for the unperturbed (*α* = 0) and perturbed (*α* = 0.1°) PhCs. Two fundamental bands of the unperturbed PhC are labeled as TE_1_ and TE_2_. These modes below the sub‐light line (dashed black line) in the gray regions behave as GMs and are localized in the transverse direction across the slab due to TIR. The GMs of the unperturbed PhC at the high‐symmetry points (X, M, and Y) of the TE_1_ and TE_2_ bands are marked with hollow upward and downward triangles, respectively.

**FIGURE 1:**
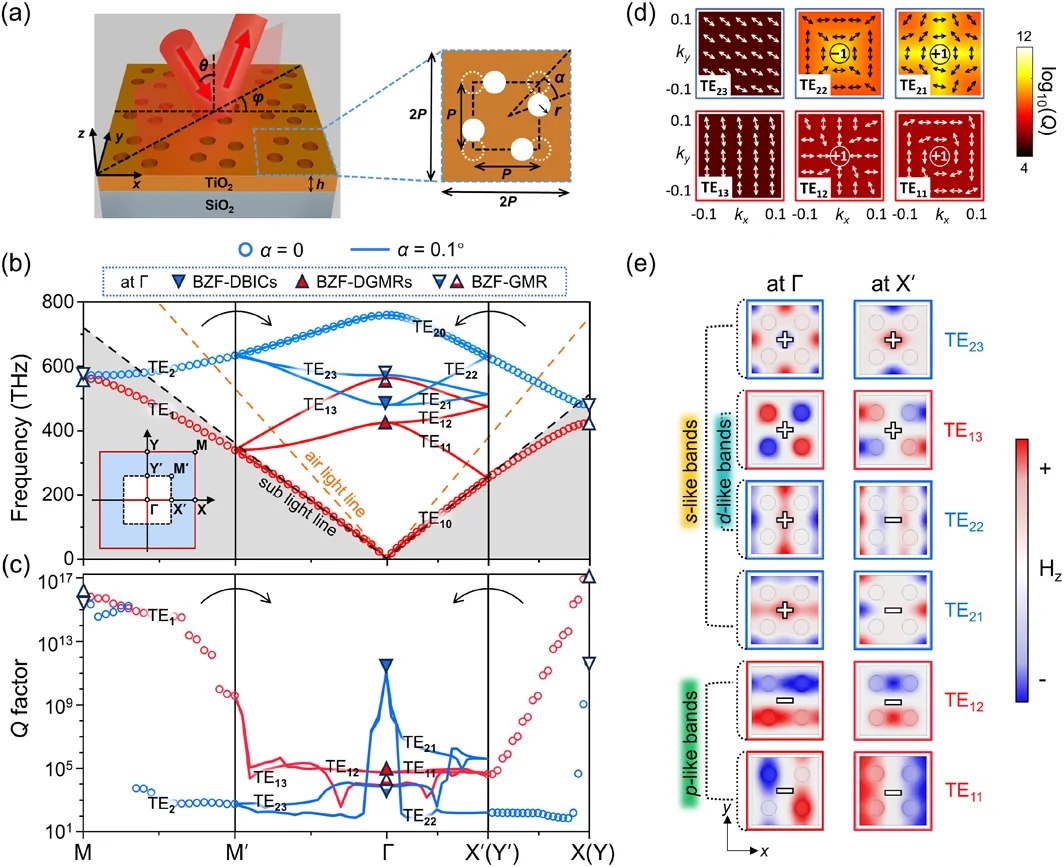
Working principle of the BZF through the twisted PhC. (a) Schematic of a twisted TiO_2_ PhC slab with thickness *h* on a SiO_2_ substrate under the light illumination with incident angle *θ* and azimuthal angle *φ*. The radius of the air hole is *r*, and the period of the primitive unit cell is *P*. The enlarged view indicates the structural parameters of the twisted PhC. (b) Calculated TE band structures for the unperturbed (*α* = 0) and perturbed (*α* = 0.1°) PhCs are plotted with hollow circles and solid lines, respectively. The dashed yellow and black lines indicate the light lines of the unperturbed PhC in air and substrate, respectively; GMs are in the regions highlighted in gray. The GMs of the unperturbed PhC at the Brillouin zone boundaries are marked with hollow upward and downward triangles for the TE_1_ and TE_2_ bands, respectively. Introducing a twist perturbation folds these bands to the Γ point. The red and blue fully filled triangles at the Γ point indicate the BZF‐induced DGMRs and DBICs, respectively. The red (blue) half‐filled triangle at the Γ point indicates the BZF‐induced GMR. The perturbed bands are labeled as TE_*mn*_, where *m* represents the corresponding band of the unperturbed PhC, *n* = 1, 2, and 3 indicate the bands with folding, whereas *n* = 0 indicates the bands without folding. The inset figure illustrates the corresponding shrinkage of the FBZ due to the introduction of the perturbation. (c) Simulated *Q*‐factor distributions of the bands for the unperturbed and perturbed PhCs. The hollow circles and solid lines show the *Q* factors of the bands for the unperturbed and perturbed PhCs. (d) Eigenpolarization maps of the BZF‐induced bands of the perturbed PhC. (e) Eigenmode distributions at the Γ and X′ points of the BZF‐induced bands of the perturbed PhC in the *x*‐*y* plane, where “+” and “−” indicate the mode profiles with and without the *C*_2_ symmetry, respectively. Three types of bands (*s*‐like, *p*‐like, and *d*‐like) are generated due to the BZF.

Besides the bands without folding (TE_10_ and TE_20_), the introduction of a twist perturbation reduces the symmetry of the structure (*C*_4*v*_ to *C*_4_) and folds all the high‐symmetry points of the TE_1_ (TE_2_) bands to the Γ point, resulting in the generation of three new bands: TE_11_, TE_12_, and TE_13_ (TE_21_, TE_22_, and TE_23_). These three new bands arise from the shrinkage of the FBZ of the PhC across all high‐symmetry directions, which contrasts with previous studies [39, 40, 41, 42, 43, 44, 45], where only one or two new bands are produced when the FBZ shrinks along a specific direction in momentum space. Particularly, for the range Γ‐X′(Y′) in momentum space, the TE_11_ (TE_22_) bands are generated due to the BZF of the unperturbed PhC along the Γ‐X(Y) direction; the TE_12_ and TE_13_ (TE_21_ and TE_23_) bands are generated due to the BZF of the unperturbed PhC along the Y(X)‐M direction (see Supporting Information S1: Figure S1). The TE_11_ and TE_12_ (TE_21_ and TE_22_) bands are degenerate at the Γ point as the *X* and *Y* points coincide at the Γ point after the BZF. The TE_11_ and TE_12_ (TE_21_ and TE_22_) bands are degenerate along the Γ‐M′ direction as the intracellular hopping and intercellular hopping are approximately equal for the PhC under small perturbations according to the two‐dimensional (2D) Su–Schrieffer–Heeger (SSH) model [56]. Figure 1c illustrates the *Q*‐factor distributions of the bands for the unperturbed and perturbed PhCs. According to Figure 1c, at the Γ point, the degenerate TE_11_ and TE_12_ bands are labeled as degenerate guided‐mode resonances (DGMRs) as they have a finite *Q* factor at the Γ point, whereas the degenerate TE_21_ and TE_22_ bands are labeled as degenerate BICs (DBICs) as they have an infinite *Q* factor at the Γ point. Note that the *Q* factors of the TE_1_ and TE_2_ of the unperturbed PhC vary abruptly at the crossing points with the sub‐light line, where the leaky modes are transformed into GMs in momentum space.

To characterize the topological features of the PhC in the far field, the in‐plane far‐field polarization vector is introduced as follows [57]:

(1) $$\left(c_{x},c_{y}\right)=\frac{1}{\iint_{V}dxdy}\iint_{V}e^{ik_{x}x+ik_{y}y}E\left(x,y\right)dxdy,$$

where “*V*” denotes a single unit cell in PhCs. (*k*_*x*_, *k*_*y*_) and *E* are the in‐plane wave vector components and the far‐field electric‐field vector, respectively. The Stokes parameters (*S*_*0*_, *S*_*1*_, *S*_*2*_, *S*_*3*_) can be obtained as follows: $S_{0}={\left|c_{x}\right|}^{2}+{\left|c_{y}\right|}^{2}$, $S_{1}={\left|c_{x}\right|}^{2}-{\left|c_{y}\right|}^{2}$, $S_{2}=2\text{Re}\left({c_{x}}^{*}\cdot c_{y}\right)$, and $S_{3}=2\text{Im}\left({c_{x}}^{*}\cdot c_{y}\right)$. The topological charge *q* of a polarization singularity in momentum space can be defined as follows:

(2) $$q=\frac{1}{2\pi }\oint_{L}dk_{\|}\cdot \nabla_{k_{\|}}\phi^{\left(n\right)}\left(k_{\|}\right),$$

where *L* is a loop in the counterclockwise direction around the polarization singularity in momentum space, *k*_||_ = (*k*_*x*_, *k*_*y*_) is the in‐plane wave vector. $\phi^{\left(n\right)}\left(k_{\|}\right)=\arg\left(S_{1}^{\left(n\right)}+iS_{2}^{n}\right)/2$ is the orientation angle of the major axis of the polarization ellipse determined by $\left(c_{x}^{\left(n\right)},c_{y}^{\left(n\right)}\right)$, where *S*_*i*_ is the *i*th Stokes parameter. Figure 1d presents the eigenpolarization maps for the BZF‐induced bands TE_11_, TE_12_, and TE_13_ (TE_21_, TE_22_, and TE_23_) in the perturbed PhC. Here, the TE_13_ and TE_23_ bands exhibit a low *Q* factor due to their leaky‐mode features, resulting in linearly polarized far‐field radiation without polarization singularities. In contrast, the TE_11_ and TE_12_ bands, though also low‐*Q* bands due to leakage, each possess a topological charge of +1 because of their degeneracy. Meanwhile, the TE_21_ and TE_22_ bands show ultrahigh *Q* factors at the Γ point, a consequence of their BIC‐related nonradiative characteristics. Notably, the TE_21_ band has a topological charge of +1, and the TE_22_ band has a charge of −1.

To distinguish the BZF‐induced bands due to the twist of the PhC, Figure 1e illustrates the eigenmode distributions of these bands at the Γ and X′ points in the *x*‐*y* plane, where “+” and “−” indicate the mode profiles with and without *C*_2_ symmetry, respectively. As can be seen in Figure 1e, the bands associated with BICs (TE_21_ and TE_22_) exhibit opposite parity at the Γ and X′ points, whereas the bands associated with GMRs (TE_11_, TE_12_, TE_13_, and TE_23_) have the same parity at the Γ and X′ points. According to the symmetry of the eigenmodes, the *C*_4*v*_ point group has four one‐dimensional (1D) irreducible representations, A_1_, A_2_, B_1_, and B_2_, and one two‐dimensional (2D) irreducible representation, *E*, where “1D” implies that the eigenmode is not degenerate and “2D” implies that the eigenmode is doubly degenerate [58, 59]. The *s* band is characterized by the A_1_ modes, which are invariant under both *C*_2_ (180° rotation) and *C*_4_ (90° rotation) operations. The *p* band is characterized by the doubly degenerate *E* modes, which reverse their form under *C*_2_ rotation. The *d* band is characterized by B_1_ (B_2_) modes, which are invariant under *C*_2_ rotation but reverse their form under *C*_4_ rotation. For the twisted PhC with *C*_4_ point‐group symmetry, the TE_21_ and TE_23_ bands can be classified as *s*‐like bands, the TE_11_ and TE_12_ bands can be classified as *p*‐like bands, and the TE_13_ and TE_22_ bands can be classified as *d*‐like bands. The *p*‐like band is degenerate at the Γ point as the *E* mode has two Bloch waves, and the relevant modes can be classified as $p_{+}=p_{x}+ip_{y}$ and $p_{-}=p_{x}-ip_{y}$ modes, which are denoted as the (pseudo‐) spin‐up and spin‐down states of the *p* bands. By altering the twist angle of the PhC, it is possible to dynamically control the intraband and interband interactions among the *s*‐like and *p*‐like bands arising from the folding of the FBZ across all high‐symmetry directions.

## 3 Results

### 3.1 Evolution of BZF‐Driven Band Flips Based on BICs

To investigate the details of the interaction among the *s*‐like and *p*‐like bands, the evolution of the BZF‐driven bands is illustrated as a function of the twist angle in momentum space, as shown in Figure 2. Here, we define the perturbation factor *β* = *α*/90° to quantify the magnitude of the twist angle's effect on the perturbation of the PhC. Figure 2a illustrates the evolution of the *Q* factors of the BZF‐driven bands with different twist angles. For relatively small perturbations with (*α*, *β*) ≤ (20°, 0.22), the *Q* factors of the TE_11_ and TE_12_ bands associated with the BZF‐DGMRs exhibit flat curves fitted as *Q* = *Q*_0_/*β* ^2^ due to their leaky‐mode features [59, 60], where *Q*_0_ is a constant determined by the mode and structural design and remains independent of *α*, whereas the *Q* factors of the TE_21_ and TE_22_ bands associated with the BZF‐DBICs show an inverse‐square dependence on the normalized wave vector component *k* fitted as *Q* = *Q*_0_/(*β* ^2^ *k* ^2^), and they diverge to infinity at the Γ point due to the nonradiative features of BICs. However, under a large perturbation of (*α*, *β*) = (40°, 0.44), a reversal in modal properties occurs between the TE_11_ and TE_21_ bands. Specifically, the TE_11_ band transitions from a GMR to a BIC, whereas the TE_21_ band changes from a BIC to a GMR. Additionally, the BZF‐driven bands exhibit an inverse quadratic relationship with the perturbation factor *β* for both the Γ‐X′ and Γ‐M′ directions (see Supporting Information S1: Figure S2).

**FIGURE 2:**
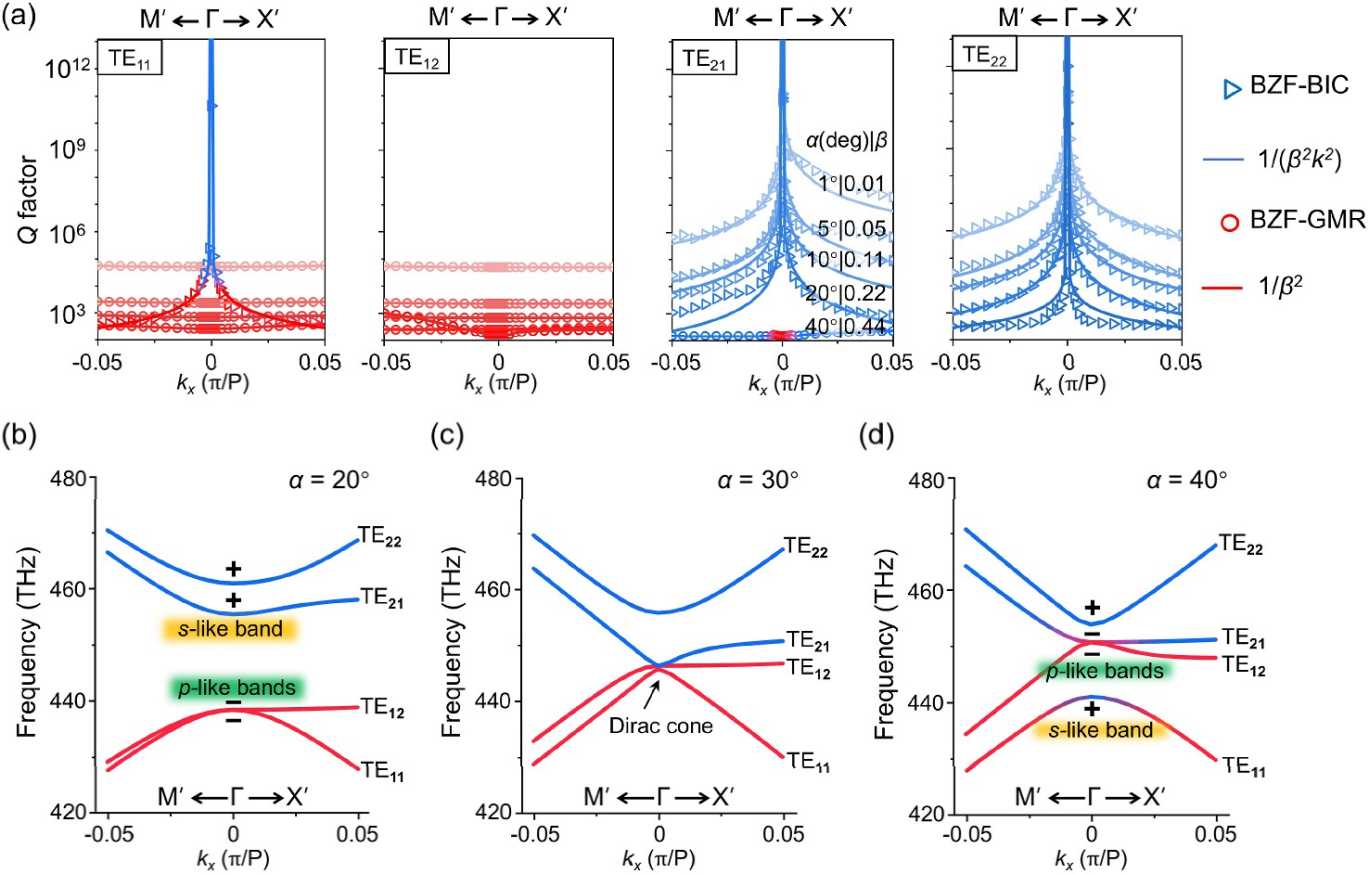
Evolution characteristics of the BZF‐driven bands of the twisted PhC based on BICs. (a) Evolution of the *Q* factor of the BZF‐driven bands with different perturbation factors *β*. For *β* ≤ 0.22, BZF‐DGMRs (TE_11_, TE_12_) show flat *Q* curves fitted as *Q* = *Q*_0_/*β* ^2^; BZF‐DBICs (TE_21_, TE_22_) show sharp *Q* curves fitted as *Q* = *Q*_0_/(*β* ^2^ *k* ^2^). At *β* = 0.44, the *Q* factors of the TE_11_ and TE_21_ bands exchange around the Γ point. Evolution of the BZF‐driven bands at (b) *α* = 20°, (c) *α* = 30°, and (d) *α* = 40°. The positive and negative signs represent the parity of the BZF‐driven bands at the Γ point. The BZF‐DBICs split at the Γ point at *α* = 20°; at *α* = 30°, a Dirac point is induced due to the accidental degeneracy of the *p*‐like (TE_11_ and TE_12_) bands and the *s*‐like (TE_21_) band at the Γ point; at *α* = 40°, the Dirac cone vanishes accompanied by topological band flips between the *s*‐like and *p*‐like bands.

To reveal the mechanism of the *Q*‐factor reversal, the evolutions of the BZF‐driven bands at different twist angles are illustrated in Figure 2b–d. For moderate perturbations with *α =* 20°, the DBICs associated with the TE_21_ and TE_22_ bands split, whereas the DGMRs associated with the TE_11_ and TE_12_ bands maintain degeneracy at the Γ point as these *p*‐like modes can be reorganized as a pair of doubly degenerate modes connected by time‐reversal operation [56]. Increasing the twist angle to 30° results in a band structure with a triply degenerate point at the Γ point, and an accidental‐degeneracy‐induced Dirac cone (ADC) is achieved due to the accidental degeneracy of the *p*‐like (TE_11_ and TE_12_) bands and the *s*‐like (TE_21_) band at the Γ point, where two linearly dispersive bands intersect at the Dirac‐point frequency of 446.1 THz, with an additional flat band along the Γ‐X′ direction. The dispersions are qualitatively similar to those of PhCs with triangular lattice symmetry [61]. Further increasing the twist angle to 40° breaks the triply degenerate bands linked to the doubly degenerate *p*‐like bands and the *s*‐like band, resulting in the disappearance of the ADC. This is accompanied by topological band flips, manifested as a parity exchange between the TE_11_ and TE_21_ bands, where their odd (−) and even (+) symmetries at the Γ point reverse across the Dirac point. The topological band flipping also results in a transition in the topological phase of the bandgap, with the Zak phase switching from 0 to π (see Supporting Information S1: Figure S3). According to the selection rule for Dirac points [62, 63, 64], the linear and conical dispersion associated with Dirac‐like points appears at degeneracies in momentum space among bands with different parities. The *p*‐like and *s*‐like bands in the twisted PhC possess opposite parities. Therefore, the merging of the *p*‐like and *s*‐like bands at the Γ point gives rise to the Dirac‐like cone. It should be noted that the parity exchange between the TE_11_ and TE_21_ bands is intrinsically linked to the transfer of the +1 topological charge between them at the Γ point. Specifically, the transfer of the +1 topological charge is accompanied by a mode transformation: The TE_11_ band's GMR mode converts into the TE_21_ band's BIC mode, and vice versa (see Supporting Information S1: Figure S4). Additionally, if the circular holes are twisted along a circular trajectory with a fixed distance from the center of the unit cell, the band evolution exhibits similar features, except for the emergence of an additional Dirac cone associated with the TE_22_ band (see Supporting Information S1: Figure S5).

To demonstrate the mode transformation associated with the BZF, Figure 3a plots the evolution of the eigenfrequencies of the TE_11_ and TE_21_ bands at the Γ point versus the twist angle *α*. It shows that at the intersection point of the ADC with (*α*, *f*) = (30°, 446.1 THz), the two bands exchange their BIC and GMR modes, and the regions associated with the *s*‐like and *p*‐like bands reverse due to the band flips in twisted PhCs. Figure 3b,c shows the calculated reflection responses of twisted PhCs with *α* equal to 25° and 35°, respectively; the incident light is slightly obliquely incident (*θ* = 3°) so as to excite the resonance of the BZF‐BIC mode. The insets in the figures are the magnetic‐field distributions of the resonant modes associated with the TE_11_ and TE_21_ bands. The near‐field pattern of the resonant mode of the TE_11_ band is similar to that of the TE_21_ band due to their band flips. To characterize the resonant modes of the TE_11_ and TE_21_ bands, the far‐field scattering distributions of the multipoles of twisted PhCs are investigated, as shown in Figure 3d,e. Here, the resonances associated with the TE_11_ and TE_21_ bands are decomposed into radiating contributions from multipoles, including electric dipoles (EDs), magnetic dipoles (MDs), toroidal dipoles (TDs), electric quadrupoles (EQs), and magnetic quadrupoles (MQs) according to the multipole decomposition theory (see Supporting Information S1: Note 6). The results show that at *α* = 25°, the TE_11_‐band resonance is dominated by a hybrid TD‐MQ mode, whereas the TE_21_ band is dominated by an MD mode. Upon the band flips at *α* = 35°, these dominant multipole modes reverse, further confirming the associated mode transformation after the band flips.

**FIGURE 3:**
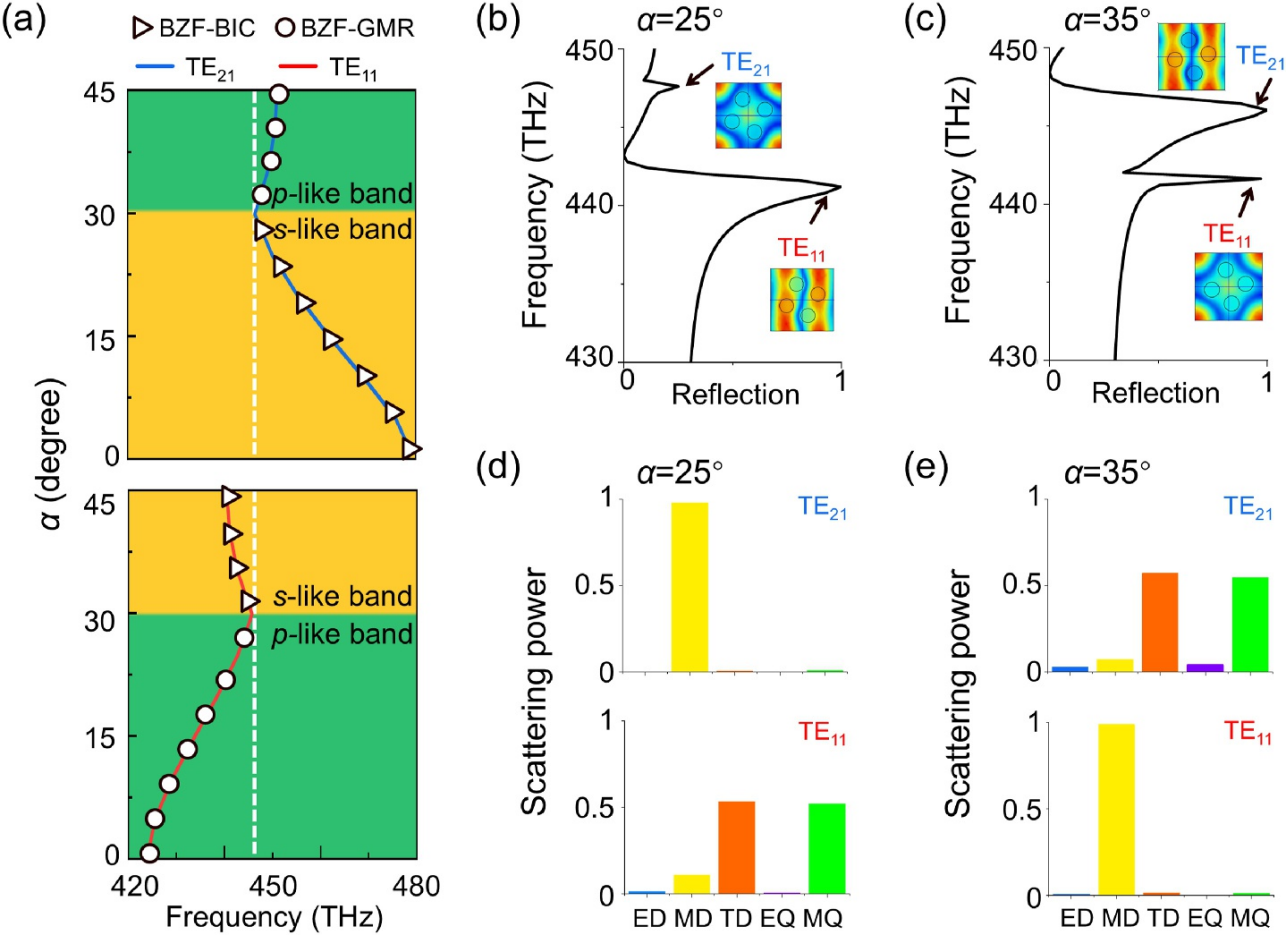
Characterization of the mode transformation between the TE_11_ and TE_21_ bands. (a) Evolution of the eigenfrequency of the TE_11_ and TE_21_ bands at the Γ point as the twist angle *α* is varied. The two bands switch between the BIC and GMR modes before and after the intersection point at 446.1 THz with *α* = 30°, and the regions of the *s*‐like and *p*‐like bands reverse due to the band flips. Reflection spectra of twisted PhCs with (b) *α* = 25° and (c) *α* = 35° at (*θ*, *φ*) = (3°, 0). The insets indicate the magnetic‐field distributions of the resonant modes. The normalized scattering power of the resonant modes of the TE_11_ and TE_21_ bands at (*θ*, *φ*) = (3°, 0) for twisted PhCs with (d) *α* = 25° and (e) *α* = 35°.

### 3.2 Experimental Demonstration

To verify our theoretical findings regarding the realization of BZF‐driven band flips through twisted PhCs based on BICs, we fabricated three types of TiO_2_ PhCs (each ∼500 *μm* × ∼500 *μm*) on a SiO_2_ substrate using ion‐assisted deposition (IAD), electron‐beam lithography (EBL), and inductively coupled plasma (ICP) etching processes (see Section 5.2: Sample Fabrication for more details on the fabrication). The geometrical parameters of the fabricated devices were characterized using scanning electron microscopy (SEM). Top‐view and side‐view SEM images of the devices are presented in Figure 4a, revealing that the fabricated parameters agree well with the design. Minor deviations are observed in the form of tilted sidewalls of the nanoholes, resulting from imperfections in the EBL and dry etching processes. Figure 4b shows the homebuilt setup for the measurement of reflection spectra of the fabricated devices. The incident light from a xenon lamp first passes through a polarizer (P1) to generate linear polarization. It is then focused via a lens (L1) onto the back focal plane, where the incident angle can be precisely controlled by translating L1. The light is subsequently focused onto the sample surface through an objective lens. After reflection, the beam travels upward through the same objective. A portion of the reflected light is directed to a spectrometer for recording reflection signals, whereas a CCD camera images the sample surface to locate measurement positions, and an eyepiece assists in focusing adjustments. Throughout the measurements, the polarization directions of P1 and P2 are kept parallel so as to block the scattered light from the background. Rotating polarizer P1 allows selection of either TE‐ or TM‐polarized incident light. By adjusting the sample orientation, reflection spectra can be measured along either the Γ‐X′ or Γ‐M′ direction of the FBZ.

**FIGURE 4:**
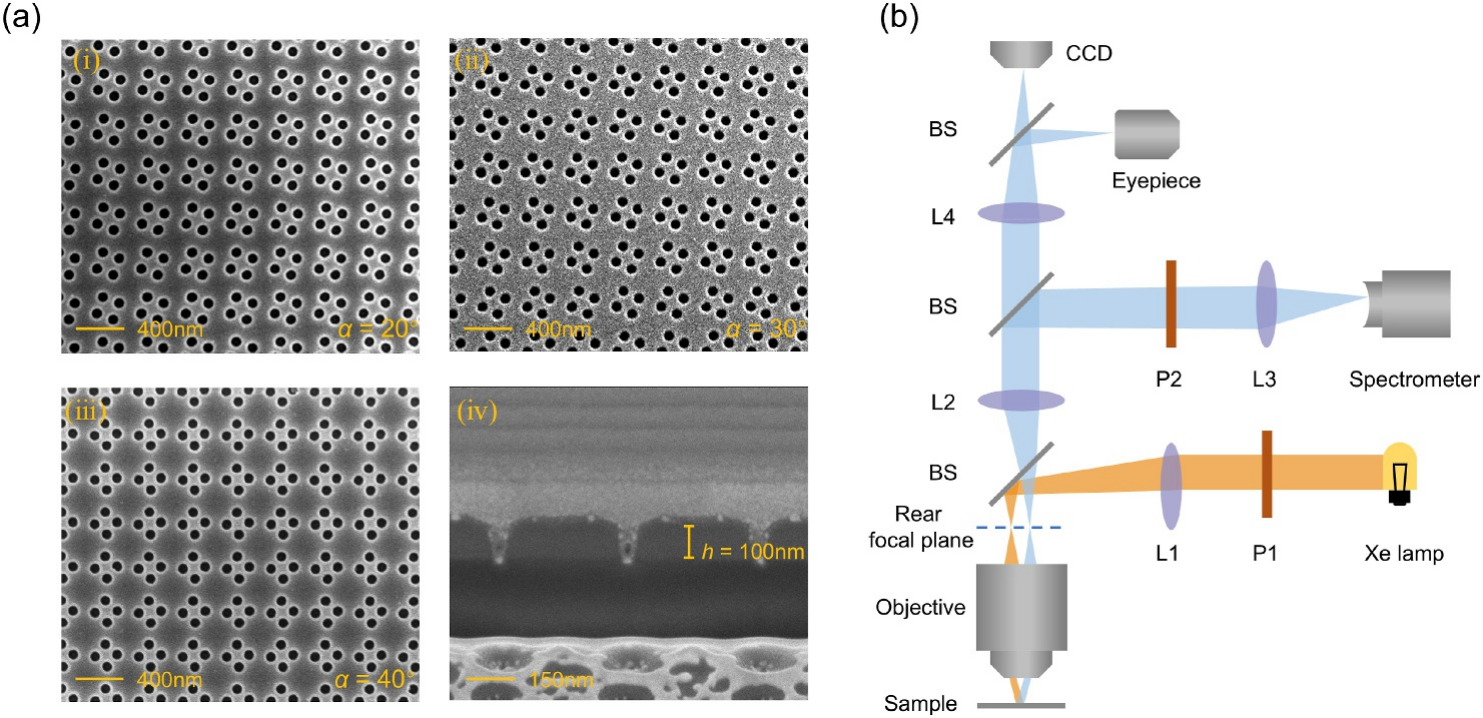
SEM images of fabricated samples and schematic of the optical measurement setup. (a) Top views for (i) *α* = 20°, (ii) *α* = 30°, (iii) *α* = 40°, and (iv) a side view of the SEM images of the samples. (b) Schematic of the measurement setup. The orange region denotes the incident light path. The blue region represents reflection from the sample.

Figure 5 illustrates the measured momentum‐space reflection spectra for three types of twisted PhCs, with dashed guidelines indicating the resonances associated with the BZF‐induced bands. TE (electric field perpendicular to the incident plane) and TM (magnetic field perpendicular to the incident plane) polarizations are chosen so as to excite all the resonances of the BZF‐induced bands. As can be seen in Figure 5b,c, at *α* = 20°, the resonances of the TE_11_ and TE_12_ bands associated with the BZF‐GMRs are degenerate at the Γ point and are excitable in momentum space. In contrast, the resonances of the TE_21_ and TE_22_ bands associated with the BZF‐BICs require oblique incidence for excitation, as their resonances vanish at the Γ point. Because of symmetry considerations regarding the even (odd) mode profiles about the incident plane, certain resonances cannot be excited under specific polarizations (see Supporting Information S1: Figure S6). Specifically, TE polarization cannot excite the resonances of the TE_12_ band along the M′‐Γ‐X′ direction and those of the TE_22_ band along the Γ‐M′ direction. Conversely, TM polarization cannot excite the resonances of the TE_11_ and TE_21_ bands along the M′‐Γ‐X′ direction, nor those of the TE_22_ band along the Γ‐X′ direction.

**FIGURE 5:**
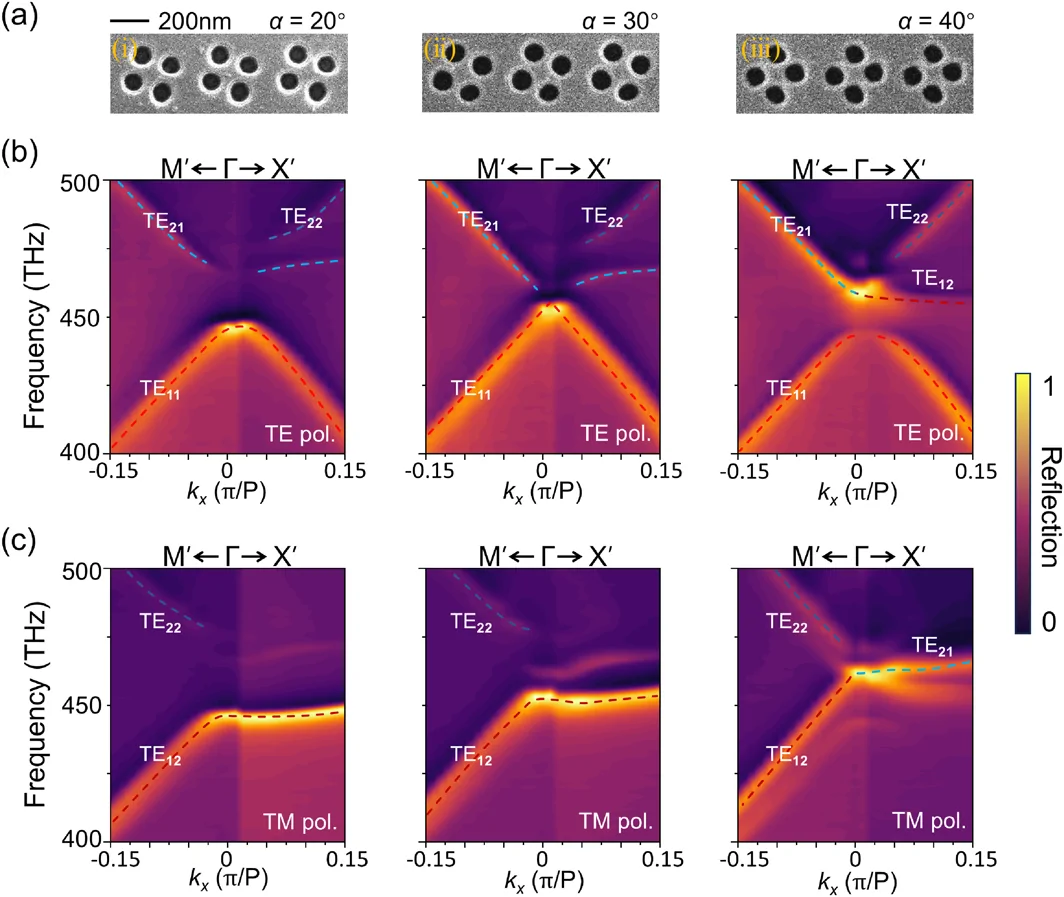
Measured reflection spectra in momentum space along the M′‐Γ‐X′ direction for three types of twisted PhCs. (a) Fabricated devices with different twist angles. 2D reflection spectra for the (b) TE and (c) TM polarizations, measured from the corresponding samples in each column. The dashed guidelines indicate the resonances associated with the BZF‐induced bands.

At the critical angle of *α* = 30°, a Dirac cone is observed due to the accidental degeneracy of the degenerate bands (TE_11_, TE_12_, and TE_21_) at the Γ point. It features two linearly dispersive bands intersecting at the quasi‐Dirac‐point frequency of 454.8 THz and a flat TE_21_ band along the Γ‐X′ direction. The Dirac point is not rigorously triply degenerate at the Γ point due to fabrication imperfections in the samples. Increasing the twist angle to 40° breaks the ADC of the triply degenerate bands and induces a topological band flip. Consequently, the GMR mode of the TE_11_ band counterintuitively converts into the BIC mode of the TE_21_ band at the Γ point due to the topological band flip. The experimental results are in good agreement with the simulation results (see Supporting Information S1: Figure S7), confirming the fundamentally important role of BZF in the band engineering of the BIC‐based PhCs.

## 4 Conclusion

In conclusion, we demonstrate the realization of BZF‐driven band flips via twisted PhCs. Introducing a twist perturbation folds all high‐symmetry points of the bands of the pristine PhC to the Γ point. As a result of the shrinkage of the FBZ across all high‐symmetry directions, each original band gives rise to three new bands, each associated with either a BZF‐BIC or a BZF‐GMR mode. The interaction among these BZF‐induced bands can be continuously tuned by varying the twist angle. Importantly, an accidental degeneracy between the *p*‐like (TE_11_ and TE_12_) and *s*‐like (TE_21_) bands at Γ establishes an ADC at a critical angle. Further twisting breaks this triply degenerate Dirac cone at the Γ point, directly initiating a topological band inversion. This transition is confirmed by parity exchanges and BIC‐GMR mode conversions within the TE_11_ and TE_21_ bands. The twist‐tuned band‐inversion process associated with an ADC is experimentally observed and shows excellent agreement with numerical simulations. Our findings suggest a novel approach for the dynamic generation and manipulation of the band structure of PhCs, providing deeper insight into the topological properties of photonic systems and their practical implications for functional optical devices such as polarization‐dependent filtering, optical sensing, and high‐harmonic generation.

## 5 Materials and Methods

### 5.1 Numerical Simulation

The eigenfrequencies of the band structures, reflection spectra, and field distributions of PhCs were calculated using the finite element method (FEM) implemented in the commercial software COMSOL Multiphysics. Reflection spectra were obtained by simulating a linearly polarized plane wave incident along the −*z* direction. Bloch periodic boundary conditions were applied along both the *x* and *y* directions, whereas perfectly matched layers (PMLs) were used in the *z* direction. The simulation mesh was refined to the finest available resolution to ensure the accuracy of the numerical results.

### 5.2 Sample Fabrication

The PhC samples were fabricated on a 600‐μm‐thick SiO_2_ substrate. Initially, a 100‐nm‐thick TiO_2_ layer was deposited on the SiO_2_ surface by the IAD. This was followed by the EBE of a 30‐nm‐thick Cr layer, which served as a hard mask. A ∼250‐nm‐thick ZEP520 A resist was then spin‐coated, soft‐baked at 180°C for 3 min, and patterned via EBL. After resist development, the patterns were transferred into the Cr film by ICP etching using a mixed gas of CHF_3_ and O_2_. The patterned Cr layer subsequently acted as a hard mask to transfer the structure into the underlying TiO_2_ film via ICP etching using a CHF_3_/SF_6_ gas mixture. Finally, the remaining Cr was removed using a Cr etchant. The procedures for sample fabrication can be seen in Supporting Information S1: Figure S8.

### 5.3 Sample Characterization

The measurement system comprises a fiber‐access source, an angle‐resolved setup, and a back‐end spectrometer. Angle resolution is achieved through mechanical rotation of the source, utilizing the Fourier space imaging technique developed by Ideaoptics Inc. China. To acquire the absolute reflection intensity of the samples, a reference aluminum mirror was first measured to normalize the incident light. All spectral measurements were conducted in a darkroom to enhance the signal‐to‐noise ratio. Additionally, topographic characterization of the samples was performed using an SEM (GeminiSEM 500, ZEISS).

## Author Contributions

**Guanhua Yang:** writing–review and editing, writing–original draft, methodology, software, data curation, formal analysis, validation, investigation. **Tian Sang:** conceptualization, methodology, investigation, writing–original draft, writing–review and editing, funding acquisition, supervision. **Leyi Zhu:** software, formal analysis. **Chenyang Wang:** methodology, validation. **Yueke Wang:** methodology, formal analysis.

## Funding

This research was supported by the National Natural Science Foundation of China (Grant 62375113).

## Conflicts of Interest

The authors declare no conflicts of interest.

## Supporting information


Supporting Information S1

## Data Availability

All data needed to evaluate the conclusions in the paper are present in the main text and the supplemental document. The datasets generated and analyzed during this study are available from the corresponding author upon reasonable request.
