# Supplementary material for: Brillouin Zone Folding‐Driven Topological Band Flips Based on BICs via Twisted Photonic Crystals
Source: Nanophotonics. 2026 Jun 9;15(11):e70164. doi: 10.1002/nap2.70164 (PMC13642103; doi:10.1002/nap2.70164)
Supplement: Supplementary file 1 [file NAP2-15-e70164-s001.docx]

Supplementary Information for

**Brillouin Zone Folding-Driven Topological Band Flips Based on BICs via Twisted Photonic Crystals**

Guanhua Yang^1,2^ | Tian Sang^1,2,*^ | Leyi Zhu^1,2^ | Chenyang Wang^1,2^ | Yueke Wang^1,2^

^1^Department of Optoelectronic Information Science and Engineering, School of Optoelectronic Information and Physical Science, Jiangnan University, Wuxi 214122, China **|** ^2^Jiangsu Provincial Research Center of Light Industrial Optoelectronic Engineering and Technology, Jiangnan University, Wuxi 214122, China

**^*^Corresponding author:** Tian Sang (sangt@jiangnan.edu.cn)

**The Supplemental Document include:**

Supplementary Note 1: BZF-induced bands along the Y(X)-M direction

Supplementary Note 2: *Q* factor distributions of the BZF-induced bands

Supplementary Note 3: Topological phase evolution characterized by Zak phase

Supplementary Note 4: Mode transformation between TE_11_ and TE_21_ bands at Γ point

Supplementary Note 5: Topological band flips under circular twisting trajectory

Supplementary Note 6: Multipole decompositions in Cartesian coordinates

Supplementary Note 7: Symmetry consideration for excitation of resonant modes

Supplementary Note 8: Simulated 2D reflection spectra

Supplementary Note 9: Sample fabrication

**Supplementary Note 1:** **BZF-induced bands along the Y(X)-M direction**

**FIGURE S1 |** **Band structures of the PhCs with and without the folding of the FBZ.** The unperturbed (*α* = 0) and perturbed (*α* = 0.1°) PhCs are plotted with hollow circles and solid lines, respectively. The dashed arrows indicate that the TE_12_ and TE_13_ (TE_21_ and TE_23_) bands are generated due to the BZF folding of the unperturbed PhC along the Y(X)-M direction.

**Supplementary Note 2: *Q* factor distributions of the BZF-induced bands**

**FIGURE S2 | *Q* factor distributions of the BZF-induced bands as functions of perturbation factor *β***. Simulated and fitting results of the *Q* factor of the (a) TE_11_ and (b) TE_12_ bands at the Γ point as functions of *β*. (c) Simulated and fitting results of the *Q* factors of the TE_21_ and TE_22_ bands at the Γ-X′ direction with (*k_x_*, *k_y_*) = (0.01, 0). (d) Simulated and fitting results of the *Q* factors of the TE_21_ and TE_22_ bands at the Γ-M′ direction with (*k_x_*, *k_y_*) = (-0.01, -0.01).

Figure S2 shows the *Q* factor distributions of the BZF-induced bands as functions of perturbation factor *β.* As can be seen in Figures S2a and S2b, for the *Q* factor of the TE_11_ and TE_12_ bands at the Γ point, their *Q* factors are decreased with the increase of *β*, which follow an inverse quadratic function relationship fitted as $Q=Q_{0}/\beta^{2}$ due to their leaky mode features[1, 2]. Notably, in Figure S2a, the *Q* factor of the TE_11_ band increases by several orders of magnitude around *β* = 0.4, which is associated with the mode transformation from the GMR to BIC due to the band flips. In Figures S2c and S2d, it can be seen that the *Q* factors of the TE_21_ and TE_22_ bands is also inversely proportional to *β*, and they show the inverse quadratic function relationship fitted as $Q=Q_{0}/(\beta^{2}k^{2})$ due to the BZF-BIC features[3]. Slight discrepancies emerge at larger *β*, primarily due to deviations from the small perturbation approximation.

**Supplementary Note 3：Topological phase evolution characterized by Zak phase**

**FIGURE S3 | Full band structure along M′−Γ−X′−M′ and eigenfield distributions before (*α* = 20°) and after (*α* = 40°) the band flips.** (a) Full band structure along M′−Γ−X′−M′ for the twisted PhCs with *α* = 20°. (b) Eigenfield distributions at Γ and X′ for the twisted PhCs with *α* = 20°. (c) Full band structure along M′−Γ−X′−M′ for the twisted PhCs with *α* = 40°. (d) Eigenfield distributions at Γ and X′ for the twisted PhCs with *α* = 40°.

Figure S3a shows the full band structure along M′−Γ−X′−M′ at a twist angle of 20°. A locally magnified plot is provided to clearly resolve the band distribution at the Γ point, where the odd and even parities of eigenfields at Γ and X′ are marked by “−” and “+” signs, respectively. The corresponding eigenfield distributions are displayed in Figure S3b. According to the calculation formula of the Zak phase [4]:$P_{i}=\text{ }\pi\left(\sum_{n} q_{i}^{n}\mathrm{mod} 2\right), (-1)^{q_{i}^{n}}=\frac{\eta_{n}(X_{i})}{\eta_{n}(\Gamma)}$. Where *i* denotes the *x*/*y* direction of the crystal, X*_i_* represents the high-symmetry point (X′) in the Brillouin zone, *η_n_*(**k**) is the eigenstate parity of the *n*th band at point **k**, and the summation runs over all occupied bands. The Zak phase of the bandgap is calculated to be 0, corresponding to a topologically trivial phase.

Figure S3c presents the full band structure along M′−Γ−X′−M′ at a twist angle of 40°. A local magnification at the Γ point is also included, and the parities at high-symmetry points are marked in the same way. The corresponding eigenfield distributions are shown in Figure S3d. The Zak phase of the bandgap is calculated to be π, corresponding to a topologically nontrivial phase.

These results clearly demonstrate that during the topological band flipping induced by the twist angle, the topological phase of the bandgap changes essentially, with the Zak phase switching from 0 to π.

**Supplementary Note 4: Mode transformation between TE_11_ and TE_21_ bands at Γ point**

Figure S4 displays the distributions of the far-field polarization and *Q* factor before (*α* = 20°) and after (*α* = 40°) the band flip, with the color bar indicating the *Q* factor. As shown in Figure S4a, at *α* = 20°, the TE_11_ and TE_12_ modes exhibit GMR characteristics, manifested as a finite *Q* factor and a flat profile in *k*-space. Their far-field polarization directions are mutually perpendicular owing to their degenerate nature. In contrast, the TE_21_ and TE_22_ modes behave as BICs, featuring an infinite *Q* factor at the Γ point. However, following the topological band flip at *α* = 40° (Figure S3b), the GMR mode originally on the TE_11_ band transforms into the BIC mode on the TE_21_ band, while the BIC mode on the TE_21_ band converts into the GMR mode on the TE_11_ band. Additionally, the far-field polarizations of the TE_12_ and TE_21_ modes are orthogonal at *α* = 40°, consistent with their degenerate behavior after the band flips shown in Figure 2(d).

**FIGURE S4 | Distributions of the far-field polarization and *Q* factor before (*α* = 20°) and after (*α* = 40°) the band flips.** Far-field polarization distributions of the BZF-induced bands for the twisted PhCs with (a) *α* = 20° and (b) *α* = 40°, the color bar represents the *Q* factor.

**Supplementary Note 5：Topological band flips under circular twisting trajectory**

**FIGURE S5 | Topological band flips tuned by the twist angle under the circular trajectory of the metasurface.** (a) Schematic illustration of the circular twisting trajectory of the metasurface. Band distributions of the metasurface at twist angles of (b) 25°, (c) 28.5 °, (d) 29.5°, (e) 30.5°, and (f) 35°. Q-factor distributions of the bands and eigenmode distributions at the Γ point for the metasurface at twist angles of (g) 25°, (h) 29.5°, and (i) 35°.

In Figure S5a, we replace the twisting trajectory in the main text with a circular trajectory centered at the unit cell, so that the hole-hole distance and the hole-unit-cell center distance remain unchanged. In the series of band distribution diagrams (Figures S5b–f) with varying twist angles, at 25°, TE_11_/TE_12_ are degenerate at the Γ point, while TE_21_/TE_22_ remain separate. When the angle increases to 28.5°, three bands TE_11_/TE_12_/TE_21_ form an accidentally degenerate Dirac cone. Further increasing the twist angle breaks the Dirac cone and realizes the topological band flip between TE_11_ and TE_21_. When the angle increases to 30.5°, three bands TE_12_/TE_21_/TE_22_ form an accidentally degenerate Dirac cone. Further increasing the twist angle breaks the Dirac cone and realizes the topological band flip between TE_12_ and TE_22_. As observed in Figures S5g–i, both topological band flips are accompanied by the reversal of parity and Q-factor characteristics. Although the topological band flips can also be generated by using circular twisting trajectory, the two-band flip process involving two Dirac cones is restricted to twist angles within 2°, thereby raising the fabrication difficulty of the PhCs.

**Supplementary Note 6 Multipole decompositions in Cartesian coordinates**

According to multipole decomposition theory [5, 6], the scattering power from optical metasurfaces can be decomposed into contributions from various multipoles. These include electric dipoles (ED), magnetic dipoles (MD), toroidal dipoles (TD), electric quadrupoles (EQ), and magnetic quadrupoles (MQ). The corresponding polar moments can be expressed as:

$$P=\frac{1}{i\omega}\int jd^{3}r$$

$$M=\frac{1}{2c}\int\left(r\times j\right)d^{3}r$$

$$T=\frac{1}{10c}\int\left[\left(r\cdot j\right)r-2r^{2}j\right]d^{3}r$$

$$Q_{\alpha,\beta}^{\left(e\right)}=\frac{1}{i2\omega}\int\left[r_{\alpha}j_{\beta}+r_{\beta}j_{\alpha}-\frac{2}{3}\left(r\cdot j\right)\delta_{\alpha,\beta}\right]d^{3}r$$

$$Q_{\alpha,\beta}^{\left(m\right)}=\frac{1}{3c}\int\left[\left(r\times j\right)_{\alpha}r_{\beta}+\left(r\times j\right)_{\beta}r_{\alpha}\right]d^{3}r$$

where ***P***, ***M***, ***T***, $Q_{\alpha,\beta}^{\left(e\right)}$, and $Q_{\alpha,\beta}^{\left(m\right)}$ correspond to the contributions of ED, MD, TD, EQ, and MQ, respectively. The subscripts *α* and *β* both take values of *x*, *y*, and *z*, representing the three spatial dimensions in the Cartesian coordinate system.

When the metasurface is subjected to a harmonic excitation with an angular frequency *ω* and a time dependence of ∼exp(*iωt*), the scattering power *I* can be obtained by superimposing the scattering contributions of each multipole, and its expression is as follows:

$$I=\frac{2\omega^{4}}{3c^{3}}\left|P\right|^{2}+\frac{2\omega^{4}}{3c^{3}}\left|M\right|^{2}+\frac{2\omega^{6}}{3c^{5}}\left|T\right|^{2}+\frac{\omega^{6}}{5c^{5}}\sum_{\alpha,\beta} \left|Q_{\alpha,\beta}^{\left(e\right)}\right|^{2}+\frac{\omega^{6}}{40c^{5}}\sum_{\alpha,\beta} \left|Q_{\alpha,\beta}^{\left(m\right)}\right|^{2}+o\left(\omega\right)$$

where o(*ω*) represents the scattering contribution from higher-order multipoles, and these higher-order terms are relatively weak and can be neglected.

**Supplementary Note 7: Symmetry considerations for excitation of resonant modes**

**FIGURE S6 | Mode symmetry of the BZF-induced bands about the incident plane along the Γ-X′ and Γ-M′ directions.** The eigenmode distributions for the BZF-induced bands along Γ-X′ direction with (*k_x_*, *k_y_*) = (0.01, 0) and Γ-M′ direction with (*k_x_*, *k_y_*) = (-0.01, -0.01). The black dashed lines indicate the incident plane, with the parity of the modes with respect to this plane classified as even or odd according to their mode profiles.

Figure S6 illustrates the eigenmode distributions for the BZF-induced bands along Γ-X′ direction with (*k_x_*, *k_y_*) = (0.01, 0) and Γ-M′ direction with (*k_x_*, *k_y_*) = (-0.01, -0.01). According to the symmetry considerations [7, 8], the polarization of the incident light determines the specific PhC modes that are excited. This is because a source of a given symmetry couples only to optical modes sharing the same symmetry. In terms of field distribution, this manifests as follows: modes with odd symmetry relative to the incident plane can be excited by the TM polarization, whereas those with even symmetry relative to the incident plane can be excited by the TE polarization. Therefore, for the twisted PhC with *α* = 20°, along the Γ-X′ direction, the TE_11_, TE_21_, and TE_22_ bands can be excited by the TE polarization, and the TE_12_ band can be excited by the TM polarization; however, along the Γ-M′ direction, the TE_11_ and TE_21_ can be can be excited by the TE polarization, and the TE_12_ and TE_22_ can be excited by the TM polarization. In the case of *α* = 40°, the BZF-induced bands also be selectively excited along the Γ-X′ and Γ-M′ directions according to the symmetry considerations. The selective excitation properties of the BZF-induced bands can be evident from the measured 2D reflection maps in Figure 5.

**Supplementary Note 8: Simulated 2D reflection spectra**

**FIGURE S7 | Simulated 2D reflection spectra of three types of twisted PhCs under the illumination of the (a) TE and (b) TM polarizations.**

**Supplementary Note 9: Sample fabrication**

**FIGURE S8 | Fabrication procedures of the twisted PhCs.**

**References**

1.	A. C. Overvig, S. Shrestha, and N. Yu, “Dimerized high contrast gratings,” *Nanophotonics* 7, no. 6 (2018): 1157–1168, https://doi.org/10.1515/nanoph-2017-0127.

2.	A. C. Overvig, S. C. Malek, M. J. Carter, et al., “Selection Rules for Quasi-Bound States in the Continuum,” *Physical Review B* 102, no. 3 (2020): 035434, https://doi.org/10.1103/PhysRevB.102.035434.

3.	W. Wang, Y. K. Srivastava, T. C. Tan, et al., “Brillouin Zone Folding Driven Bound States in the Continuum,” *Nature Communications* 14, no. 1 (2023): 2811, https://doi.org/10.1038/s41467-023-38367-y.

4.	F. Liu, H. Deng, and K. Wakabayashi, “Topological Photonic Crystals with Zero Berry Curvature,” *Physical Review B* 97, no. 3 (2018): 035442, https://doi.org/10.1103/PhysRevB.97.035442.

5.	T. Kaelberer, V. A. Fedotov, N. Papasimakis, et al., “Toroidal Dipolar Response in a Metamaterial,” *Science* 330, no. 6010 (2010): 1510–1512, https://doi.org/10.1126/science.1197172.

6.	Z. Sadrieva, K. Frizyuk, M. Petrov, et al., “Multipolar Origin of Bound States in the Continuum,” *Physical Review B* 100, no. 11 (2019): 115303, https://doi.org/10.1103/PhysRevB.100.115303.

7.	J. Lee, B. Zhen, S. Chua, et al., “Observation and Differentiation of Unique High-*Q* Optical Resonances Near Zero Wave Vector in Macroscopic Photonic Crystal Slabs,” *Physical Review Letters* 109, no. 6 (2012): 067401, https://doi.org/10.1103/PhysRevLett.109.067401.

8.	Z. Jing, T. Sang, S. Ouyang, et al., “Giant Absorption of Monolayer Graphene Empowered by Degenerate Quasi-Bound States in the Continuum,” *Optics Letters* 50, no. 10 (2025): 3253–3256, https://doi.org/10.1364/OL.561193.
